# Pulmonary Paragonimiasis in Native Community, Esmeraldas Province, Ecuador, 2022

**DOI:** 10.3201/eid2810.220927

**Published:** 2022-10

**Authors:** José C.N. Diaz, Mariella Anselmi, Manuel Calvopiña, Mayra E.P. Vera, Yuvy L.C. Cabrera, Javier J. Perlaza, Luz A.O. Cabezas, Christian O.R. Gaspar, Dora Buonfrate

**Affiliations:** Distrito de Salud 08D05 San Lorenzo, San Lorenzo, Ecuador (J.C.N. Diaz, Y.L.C. Cabrera, J.J. Perlaza, L.A.O. Cabezas, C.O.R. Gaspar);; Centro de Epidemiologia Comunitaria y Medicina Tropical, Esmeraldas, Ecuador (M. Anselmi);; Universidad de las Americas, Quito, Ecuador (M. Calvopiña);; Energy e Palma Energypalma SA, Montecristi, Ecuador (M.E.P. Vera);; Istituto di Ricovero e Cura a Carattere Scientifico IRCCS Sacro Cuore Don Calabria Hospital, Negrar, Verona, Italy (D. Buonfrate)

**Keywords:** paragonimiasis, native community, Paragonimus, P. mexicanus, fluke, flatworm, trematode, food-borne infection, food safety, tuberculosis and other mycobacteria, zoonoses, Esmeraldas Province, Ecuador

## Abstract

Paragonimiasis is a food-borne infection caused by several species of the *Paragonimus* fluke. Clinical manifestations can mimic tuberculosis and contribute to diagnostic delay. We report a cluster of paragonimiasis in a community in Ecuador, where active surveillance was set up after detection of the first 2 cases.

Human paragonimiasis is a foodborne disease caused by trematode worms of the genus *Paragonimus* (*1*). Several species that have different geographic distributions have been associated with human infection (*2*). Paragonimiasis is caused by ingestion of raw/undercooked freshwater crabs or crayfish infested by metacercariae of *Paragonimus* species. Thus, it is frequently reported in Asia because of cultural dietary customs (*1*,*3*). Clusters are occasionally reported in Africa (*4*) and the Americas, where cases are observed mostly in countries in Latin America (*5*). Localized infection with *P. kellicotti* trematode occurs in the United States (*1*,*5*).

In Ecuador, cases have been reported from almost all provinces, but lack of official recording by the Ministry of Health and few active surveillance surveys probably cause an underestimation of the incidence (*5*). Nevertheless, Ecuador is considered the country with the highest incidence of paragonimiasis in South America (*5*). The main trematode species known to cause paragonimiasis in Ecuador is *P. mexicanus*, although molecular characterization has not been performed extensively. Thus, information about circulating species might be incomplete (*5*,*6*).

Symptoms include fever and respiratory involvement, and most persons have a productive cough with rusty sputum or chest pain that can last from a few months to years. Thus, tuberculosis is the main condition to be ruled out in differential diagnosis (*6*,*7*). We report a public health intervention for a cluster of paragonimiasis observed during the end of 2021‒May 2022, in San Lorenzo, Ecuador.

The first 2 cases were diagnosed in laborers working on the same palm oil farm. They both reported productive cough with rusty sputum and dyspnea lasting for about 4 months (first case-patient) and for at least 4 years (second case-patient). The second patient had been tested several times for tuberculosis but was not previously tested for other causes of respiratory symptoms. Microscopic examination of acid-fast‒stained smears of sputum ruled out tuberculosis, but directly observed microscopic examination showed parasite eggs (Figure). Specimens were then sent to the Laboratory of Parasitology of the Universidad de las Americas in Quito, where diagnosis of paragonimiasis was made. Both patients confirmed the habit of eating raw freshwater crustaceans.

**Figure:**
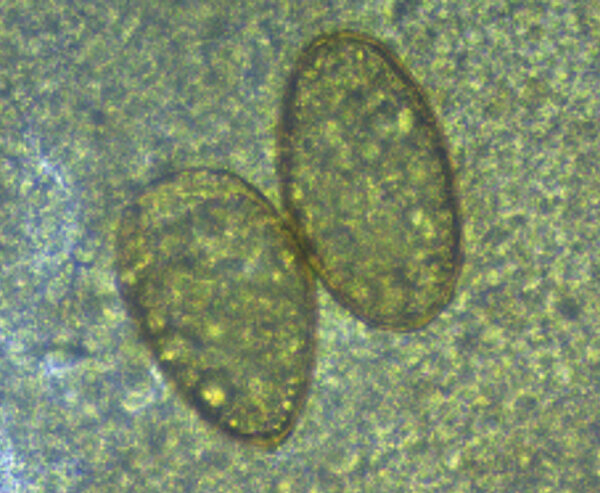
*Paragonimus* eggs from sputum from a patient in Ecuador. Eggs are yellow, elongated, have a thick shell, and are asymmetric with 1 end slightly flattened. The operculum is clearly visible at the large end and is thickened at the abopercular end. Original magnification ×40, size 80–90 μm × 45–50 μm.

After the first 2 cases, the Department of Epidemiology of San Lorenzo Health District organized a meeting with the community where the 2 case-patients lived. Aims of the intervention were to ascertain whether the infection was spreading in the community and evaluate possible control strategies. Healthcare workers collected 3 sputum samples from each of 22 persons who had compatible respiratory symptoms and 1 fecal sample from each of 36 asymptomatic persons, including family members of the positive case-patients.

*Paragonimus* eggs were found in samples from 8 persons, all from the same community (La Ceiba), except for 1 person (who lived in Balsareño but worked on the same palm oil farm as the first case-patient) (Table). Symptoms lasted for months in all persons reporting them.

**Table:** Demographic and clinical characteristics of 10 persons infected with *Paragonimus* eggs, Ecuador

Case-patient	Age, y/sex	Sample positive for eggs	Symptoms
1	24/M	Sputum	Rusty sputum and dyspnea while working, episodes of productive cough
2	32/M	Sputum	Rusty sputum and mild dyspnea, episodes of productive cough
3	27/F	Stool	None
4	20/M	Sputum	Rusty sputum
5	29/F	Stool	None
6	32/M	Sputum	Rusty sputum
7	8/M	Stool	None
8	10/M	Stool	None
9	31/F	Sputum	Rusty sputum
10	22/F	Sputum	Rusty sputum

Praziquantel was donated by the Istituto di Ricovero e Cura a Carattere Scientifico Sacro Cuore Don Calabria to the Centro de Epidemiologia Comunitaria y Medicina Tropical. This drug was administered by the physician at the local health center to infected persons at a dose of 25 mg/kg, 3 times/day for 2 days.

Our study highlights some limitations that hampered estimation of incidence of paragonimiasis in Ecuador. The first limitation is delay in diagnosis. Because knowledge about this parasite is scarce, local physicians seldom prescribe diagnostic tests that could help diagnosis, and misdiagnosis as tuberculosis can be frequent. Limited diagnostic capacity can contribute to the underestimation because the sensitivity of microscopic examination is low, in particular for persons who have mild-to-moderate disease: 30%–40% for a sputum sample and 11%–15% for a stool sample (*1*). Multiple sampling increases sensitivity (*1*), but collection of a series of specimens over time can be difficult in remote settings for cultural and logistic issues.

Although stool microscopy has low sensitivity, it can detect *Paragonimus* eggs in persons who do not have respiratory symptoms. Other diagnostic tests, such as serologic or molecular methods, are not available in Ecuador, and have been seldom used there, for research purposes (*5*,*8*). Moreover, serologic assays have been implemented mostly for other species, such as *P. westermani* and *P. kellicotti* worms (*8*,*9*), and clinical validation for *P. mexicanus* worms is lacking (*9*,*10*). Limited access to healthcare services in some remote communities can further cause late diagnosis.

Control strategies to limit human infection are hampered by the wide presence of the parasite in many domestic and wild mammals, and the complex life cycle involving 2 intermediate hosts (snail and crustacean) (*5*). Thus, health education on proper food preparation is the main intervention to reduce infections (*1*).
